# Commencements

**Published:** 1898-04

**Authors:** 


### COMMENCEMENTS.

*Atlanta Medical College.*—The fortieth annual commencement of this college was held at the Grand Opera House on the evening of March 28, 1898. Forty-six graduates were given diplomas.

Dr. T. P. Bell made the opening prayer. Dr. W. S. Kendrick, Dean, then read the faculty report.

Hon. N. J. Hammond, President of Board of Trustees, conferred the degrees, in Latin.

Rev. Henry McDonald, D.D., delivered the oration.

Col. Hammond delivered the honor medals. Dr. D. Rios Zertucche, of Mexico, received first honor; Dr. J. Jones, of South Carolina, second honor, and Dr. J. H. Johns, of South Carolina, third honor.

"Honorable mention" was received by Dr. LeRoy Napier of Georgia; Dr. W. K. Johnson of Georgia; Dr. J. M. Smith of Georgia, and Dr. A. J. Mooney of Georgia.

Music as usual.

*Southern Medical College.*—This college closed its nineteenth session with interesting exercises in the Grand Opera House on the evening of March 30th. After prayer by Bishop C. K. Nelson, the Dean, Dr. J. B. Baird, made a gratifying report of the condition of the college.

Diplomas were conferred upon twenty-seven graduates by Hon. Howard Van Epps, President of the Board of Trustees.

The address of the evening was then delivered by Judge John W. Akin, President of the Georgia Bar Association.

The first-honor medal was awarded to Dr. L. S. Hardin, of South Carolina; the second-honor medal to Dr. W. A. Tillet, of Georgia. Drs. A. Harper and C. A. Smith, both of Georgia, tied for third honor, and both received a prize. Drs. Joseph Goodwin of Tennessee, James Weddington of Georgia, Victor Lagerson of Maine, and George Turner of Georgia received honorable mention.

The music rendered by the United States Fifth Infantry band was a delightful feature of the evening.

---

THE Southern Quarantine Convention, called for Atlanta on April 12th, promises to be successful in point of attendance and in the work outlined for the convention. Some of the most prominent health officers and quarantine officials in the South have signified their intention of coming to Atlanta to attend the convention, and arrangements are being made for their reception.

Mayor Collier, chairman of the Arrangements Committee, has sent out a great many invitations to officials of the health and quarantine departments of the Southern States, and a number of responses have been received indicating that the convention will be well attended.

Surgeon-General Wyman, of the United States Marine hospital service, has stated that he will attend the convention, and other well-known officials will be here. The Committee on Arrangements has been at work and the convention will be made an interesting and instructive one.

Mayor Collier has sent out invitations to the executive officers of the Southern railroads and requested them to be present or send representatives to the convention, and this will bring a number of delegates. The roads in the Southeastern, Gulf, and Southwestern States will be invited to send representatives.

The principal object of the convention is to formulate rules for the enforcement of quarantine regulations and the conduct of handling the epidemics of contagious diseases. Other questions will be considered, principal among them that of national quarantine. Health officers from all the States interested are expected to be present. Many interesting papers will be read.

Among those expected to attend the convention are the following:

Surgeon-General Wyman of Washington; Dr. C. P. Wilkinson of Louisiana; Dr. H. B. Horlbeck of Charleston, S. C.; Dr. W. H. Sanders of Mobile, Ala.; Dr. T. S. Scales of Mobile; Dr. F. G. Ranshaw of Pensacola; Professor J. L. Ludlow of Winston, N. C.; Dr. Felix Formento of New Orleans; Dr. E. P. Griffin of Pensacola, Miss.; Dr. W. T. Oppenheimer of Virginia; Mr. J. S. B. Thompson, representing the Virginia health department; Dr. Allen Stewart of Port Royal, S. C., and many others.

---

THE Chair of Diseases of the Eye, Ear, and Throat at the Medical College of Virginia, made vacant by the death of Professor Charles M. Shields, will be filled at the annual meeting of the board of visitors of the college April 21st. All applications, accompanied by credentials, should be forwarded to Christopher Tompkins, M.D., Dean, Richmond, Va.

